# Local adaptations to frost in marginal and central populations of the dominant forest tree *Fagus sylvatica* L. as affected by temperature and extreme drought in common garden experiments

**DOI:** 10.1002/ece3.971

**Published:** 2014-02-07

**Authors:** Juergen Kreyling, Constanze Buhk, Sabrina Backhaus, Martin Hallinger, Gerhard Huber, Lukas Huber, Anke Jentsch, Monika Konnert, Daniel Thiel, Martin Wilmking, Carl Beierkuhnlein

**Affiliations:** 1 Biogeography, BayCEER, University of BayreuthBayreuth, Germany; 2 Geoecology/Physical Geography, University of LandauLandau, Germany; 3 Disturbance Ecology, BayCEER, University of BayreuthBayreuth, Germany; 4 Landscape Ecology, University of GreifswaldGreifswald, Germany; 5 Bavarian Institute for Forest Seeding and Planting (ASP)Teisendorf, Germany

**Keywords:** Common garden experiment, European beech, frost, local adaptation, minimum temperature, mortality

## Abstract

Local adaptations to environmental conditions are of high ecological importance as they determine distribution ranges and likely affect species responses to climate change. Increased environmental stress (warming, extreme drought) due to climate change in combination with decreased genetic mixing due to isolation may lead to stronger local adaptations of geographically marginal than central populations. We experimentally observed local adaptations of three marginal and four central populations of *Fagus sylvatica*L., the dominant native forest tree, to frost over winter and in spring (late frost). We determined frost hardiness of buds and roots by the relative electrolyte leakage in two common garden experiments. The experiment at the cold site included a continuous warming treatment; the experiment at the warm site included a preceding summer drought manipulation. In both experiments, we found evidence for local adaptation to frost, with stronger signs of local adaptation in marginal populations. Winter frost killed many of the potted individuals at the cold site, with higher survival in the warming treatment and in those populations originating from colder environments. However, we found no difference in winter frost tolerance of buds among populations, implying that bud survival was not the main cue for mortality. Bud late frost tolerance in April differed between populations at the warm site, mainly because of phenological differences in bud break. Increased spring frost tolerance of plants which had experienced drought stress in the preceding summer could also be explained by shifts in phenology. Stronger local adaptations to climate in geographically marginal than central populations imply the potential for adaptation to climate at range edges. In times of climate change, however, it needs to be tested whether locally adapted populations at range margins can successfully adapt further to changing conditions.

## Introduction

Projecting range shifts in response to rapid climate change has become an important topic of biogeographical research (e.g., Thomas et al. 2004; Thuiller et al. 2005). Most approaches, however, neglect phenotypic and genetic variation within the range of the species. For widespread species, the incorporation of intraspecific variability can have drastic effects on the results of such projections (Oney et al. 2013). This intraspecific variability is often expressed in local adaptations to climate and other environmental factors such as soil types (Kuser and Ching 1980; Joshi et al. 2001; Hufford and Mazer 2003; McKay et al. 2005; Bennie et al. 2010; Kreyling et al. 2012a,b2012b; Thiel et al. 2014). Local adaptation can be defined as the higher fitness of local individuals at their home site compared with that of nonlocal individuals of the same species (Biere and Verhoeven 2008). If performance of populations is correlated with environmental conditions at the origin of the population (e.g., drought tolerance of populations in common garden trials being related to summer dryness at the origin of the populations, Thiel et al. 2014), this can be interpreted as a test of local adaptation. However, case studies imply that such local adaptations to climate are species specific and may even be negligible in some species and environmental parameters (Macel et al. 2007; Beierkuhnlein et al. 2011; Weisshuhn et al. 2011).

*Fagus sylvatica* is the dominant native forest tree of Central Europe and covers a wide range of environmental conditions from southern Sweden to the Italian mountains and from northern Spain to Bulgaria (Leuschner et al. 2006). Frost in winter and spring is considered to determine its northern and northeastern range limit (Bolte et al. 2007). Frequency of such cold extremes is likely decreasing with global warming, their intensity and duration, however, may generally not decrease within this century (Kodra et al. 2011). With regard to late spring frost, the earlier onset of the vegetation period may even lead to an increased risk of late frost damage (Augspurger 2013).

Frost tolerance of temperate plant species fluctuates over the course of the year. During acclimation in autumn, the plants protect cellular membranes by accumulations of soluble carbohydrates, hydrophilic polypeptides, antioxidants, and chaperones (Thomashow 1999). Triggers for this cold hardening are low, nonfreezing temperatures and shortening photoperiods (see Basler and Koerner 2012 for species-specific sensitivities to these cues). Populations within a species range differ in their frost tolerance, with populations from warmer origins investing fewer resources into frost protection (Kreyling et al. 2012c). Consequently, *F. sylvatica* populations from warmer origins are less tolerant against winter frost than populations from colder sites (Visnjic and Dohrenbusch 2004). This finding also holds true with regard to late spring frost tolerance (Kreyling et al. 2012a). However, specific phenological behavior, that is, the timing of bud burst and leaf development, may be more important than differences in physiological cryoprotection to explain these variations in spring (Kreyling et al. 2012a). There is ample evidence for persistent phenological differences between populations in the target species of this study (e.g., von Wuehlisch et al. 1995; Visnjic and Dohrenbusch 2004), stemming from common garden experiments and provenance trials which assemble populations from different parts of the species range at a specific experimental site.

Frost tolerance depends on preceding temperature, and it has been suggested that plants grown under generally warmer conditions can lose physiological adaptations to frost (Eccel et al. 2009). Paradoxically, this could even lead to more frost damage in a warmer, yet more variable future climate (Augspurger 2013). Other climate parameters can further affect frost tolerance, for example, frost tolerance increasing with preceding water stress (Blodner et al. 2005; Kreyling et al. 2012c), which can be explained by the physiological similarity of cell damage among drought and frost, both causing exsiccation of cells. While these mechanisms are generally well established, differences in the sensitivity among populations are less well understood while being clearly relevant for projections of the behavior of populations in the face of climate change.

Local adaptation may be particularly important at range limits, where the selective pressure of climatic conditions on the individuals of a species' population is usually stronger than in its range centers and where genetic mixing may be limited due to geographic isolation of the populations (Choler et al. 2004; Kawecki 2008; Paul et al. 2011). Consequently, local adaptations are more likely to develop and may be more pronounced in marginal (i.e., geographically isolated populations at range margins sensu Tigerstedt 1973) than in central populations. *Ilex dumosa*, for instance, shows strongest local adaptation, that is, superior performance of a marginal population in its native environment versus worst performance of this population in other environments in comparison with other populations from the range center (Coulleri 2010). Recently, it has been shown that microevolutionary adaptation to drought can occur within short geographic distances in our target species *F. sylvatica*, yet such adaptations can further easily spread via gene flow (Pluess and Weber 2012). In line with these findings, evidence suggests that speciation events primarily occur at range margins (e.g., Hardie and Hutchings 2010; Thompson and Rich,^2011^). While local adaptations may be beneficial for species ranges in a stable environment, rapid climate change may pose threats to locally adapted and genetically isolated marginal populations as they might lack the potential for further adaptations (Nunes et al. 2009). Conversely, strong selection and reduced gene flow in marginal populations may also enable quick adaptations to changing climate in some populations (Jump et al. 2006), although other populations may fail. It has been shown that over the Pleistocene climate oscillations speciation occurred mainly at range edges while lineages at range centers were mostly stable (Budd and Pandolfi 2010). In order to project potential responses of species to climate change, understanding the role of local adaptation in marginal versus central populations therefore appears important.

Here, we quantified the frost tolerance of four central and three marginal populations of *F. sylvatica* in common garden experiments at two experimental sites differing in mean temperature. We hypothesized that (1) frost tolerance depends on the climatic origin of populations, with decreasing frost tolerance in populations from warmer climates (local adaptation) and local adaptation to frost being stronger in marginal than in central populations. In addition, we hypothesized that (2) continuous warming reduces frost tolerance and that (3) differences between all populations in late spring frost tolerance are stronger than differences in their mid-winter frost tolerance and can be explained by phenological differences in bud break. Finally, we tested whether (4) preceding water stress increases frost tolerance independently from phenological differences.

## Methods

This research on the frost tolerance of central and marginal populations of *F. sylvatica* took place in common garden experiments at two contrasting sites: a cold site (Bayreuth, Germany, 49°55′19″N, 11°34′55″E; mean annual temperature: 8.2°C, mean coldest month temperature:−1.0°C) and a warm site located in the Upper Rhine Valley (Siebeldingen, Germany, 49°13′03″N, 8°02′47″E; mean annual temperature: 10.1°C, mean coldest month temperature: 1.0°C).

**Figure 1:**
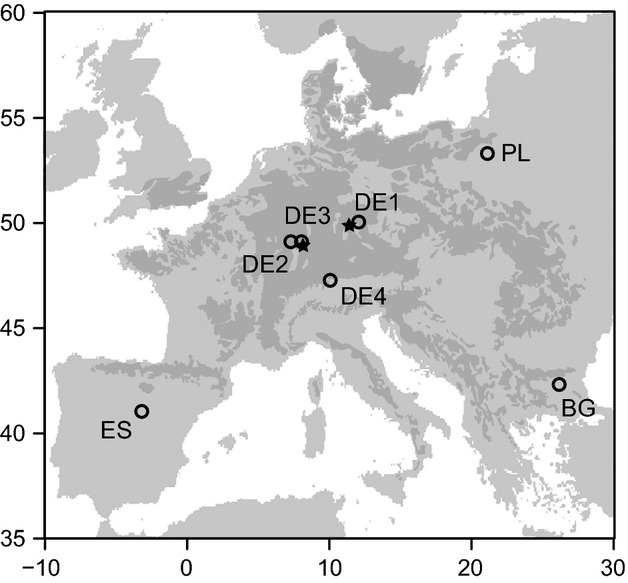
Location of the target populations within the distribution of *Fagus sylvatica* (dark gray; EUFORGEN 2009). Stars mark the locations of the two experimental sites with the cold site being located further east.

Seeds of seven populations of *F. sylvatica* were obtained in autumn 2009 from at least 20 mother trees per population (except for population DE3 were only three mother trees fructified in the year of sampling) and germinated at the Bavarian Institute for Forest Seeding and Planting (ASP) in Teisendorf, Germany, in spring 2010 in greenhouses and then grown outside under shading in common nursery substrate without additional fertilization and watered only if necessary. All seven populations stem from autochthonous populations at the center (DE1, DE2, DE3, DE4) and the southeastern (BG), southwestern (ES), and northeastern (PL) margins of the distribution range (Fig. 1, Table 1). DE3 stems from an edaphically dry site at a local limit of *F. sylvatica* distribution. DE2 originates from a nearby, but much wetter site with well-developed soils. In autumn 2010, one set of the seedlings was transported to the cold site and planted in 12-L pots (substrate: mixture of bark humus and broken limestone up to 32 mm at the volumetric rate of 1:1.6, pH_water_ = 7.7). In March 2011, the second set of seedlings was transported to the warm site and planted in 12-L pots (substrate: 50 vol-% sandy loam from local forest floor plus 50 vol-% arenaceous quartz sand, pH_water_ = 8.5). Due to these differences in experimental conditions, no direct comparison among data from both sites is undertaken. Individuals were selected randomly for each population and treatment from all living plants at planting date. Mean plant height at the start of the experiment was smaller for the Spanish population 18 cm (ES) than for the other populations which varied between 22 cm (DE4) and 25 cm (DE3). All plants were placed inside rainout shelters, which were covered with a transparent polyethylene sheet (0.2 mm, SPR5, Hermann Meyer GmbH). The lower edge of the sheets was at a height of 80 cm, and they permitted nearly 90% penetration of photosynthetically active radiation. Shading nets reduced radiation by another 30% as regeneration of *F. sylvatica* usually occurs below an open tree canopy. Plants were watered twice a week with rainwater (cold site) or groundwater (warm site) according to the local 30-year average precipitation.

**Table 1:** Site information for the populations used in this study.

Range	Code	Location	Country	Latitude	Longitude	Alt.	MAT	MAP	*T*_min_	*T*_min4_
Margin	BG	Kotel	Bulgaria	42.32724	26.17040	600	12.4	696	−2.6	6.2
Margin	ES	Montejo de la Sierra	Spain	41.04632	−3.19296	1350	10.4	512	−1.5	2.9
Margin	PL	Mragowo	Poland	53.31200	21.12000	137	6.8	667	−8.7	4.0
Center	DE1	Hengstberg	Germany	50.04800	12.06600	569	6.8	758	−5.3	1.7
Center	DE2	Johanniskreuz	Germany	49.10800	7.30000	570	7.6	900	−2.4	3.4
Center	DE3	Kalmit	Germany	49.11760	8.04380	670	7.3	700	−2.1	4.3
Center	DE4	Kempten	Germany	47.28128	10.06744	803	6.9	1457	−8.3	−1.7

Alt., elevation asl (m); MAT, mean annual temperature (°C); MAP, mean annual precipitation (mm); *T*_min_, mean minimum temperature (°C); *T*_min4_, mean minimum temperature in April (°C). Climate data derived from WorldClim (Hijmans et al. 2005).

We focused on juvenile trees in this experiment, which probably are more sensitive against frost events than older trees (Ningre and Colin 2007). However, the juvenile stage is crucial for natural regeneration of forests, and the high selective pressure of events such as frost may determine the genetic composition of future stands. Furthermore, potted plants may be more sensitive to frost damage, in particular to soil frost and root damage. Therefore, the obtained results in this study are intended for relative comparisons among populations and climate treatments.

At the cold site, two temperature treatments were fully crossed with the seven population origins in three replications with an additional nested replication of 12 individuals per population and treatment (total *n *= 504 individuals). In addition to reference conditions below the shelters, a warming manipulation took place both passively (wind shelters reducing wind speed by 70% and black floor covers vs. white floor covers) and actively (IR-radiation with approximately 30 W/m^2^), which increased mean temperature at −2 cm in the pots by 1.5°C. Minimum temperature in the control treatment reached −18.6°C (air temperature at 50 cm height) and −19.1 (soil temperature at 2 cm depth), while minimum temperature in the warming treatment reached only −17.1°C (air) and −15.8°C (soil; all values are means over six sensors). Temperature course over the full experimental period is provided in the supporting information Figure S1.

For the warm site, the population origin treatment (seven populations) was fully crossed with two precipitation treatments, that is, the local long-term average and a drought event of 36 days (starting 9 May 2011). All missing precipitation over that period was added over three consecutive days at the end of the drought manipulation (see Thiel et al. 2014 for further details). Here, we used nine individuals per population and precipitation treatment (total *n *= 126), which were kept completely randomized inside the rainout shelters. Minimum air temperature at the warm site was −15.2°C (February 7), see Figure S1 for full course of temperatures.

### Response parameters

Frost tolerance was quantified by the relative electrolyte leakage (REL) method of ex situ samples according to Kreyling et al. (2012c). The multitude of different technical protocols for REL used in the literature (freezing with or without additional solution, various freezing rates and durations, etc.) strongly limits the comparability among studies. Yet relative differences within a protocol are robust (Sutinen et al. 1992). Therefore, we stick to the interpretation of relative differences within our study and minimize the discussion of absolute values.

At the cold site, 15 lateral buds from all nested plants (*n *= 12) per population and temperature treatment were mixed per true replication (shelter) at the cold site on 31 January 2011 (*n *= 42 mixed samples: seven populations × two warming treatments × three replications). At the warm site, 15 lateral buds were sampled from each of four individuals without pooling on 6 February 2012 and 16 April 2012 (*n *= 56 samples from individual plants per date: seven populations × two precipitation treatments × four replicates). Different plants were sampled at the different dates, with all plants being destructively harvested right after bud sampling. Samples were rinsed with deionized water, cut to 0.5 cm, and mixed. Each sample was subsequently divided into seven subsamples subjected to different temperature levels (+5°C, −10°C, −20°C, −30°C, −40°C, −50°C, and −196°C [liquid N]) in manually controlled freeze boxes. Samples being wet and rate of cooling set to 5°C per hour down to −50°C prevented supercooling. After slowly cooling down to −50°C, the final subsample was immediately suspended into liquid nitrogen. Initial electrolyte leakage was determined in 16 mL 0.1% (v/v) Triton X-100 Bidest after 24 h and shaking, and the final electrolyte leakage was determined 24 h after autoclavation and shaking of the samples. Electrolyte leakage was quantified by the conductivity of the solution at 20°C measured with a WTW inolab pH/Cond 720. Blanks were analyzed throughout the procedure and used for the correction of all conductivity measurements. Frost tolerance is expressed as the LT50 for each sample, estimated by nonlinear regression of the REL versus the temperature levels using the formula by Anderson et al. (1988):


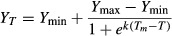
(1)

*Y*_T_ is the REL at temperature *T*,*Y*_min_ is the asymptotic value of the response variable in uninjured tissue, *Y*_max_ is the asymptotic value at maximum low-temperature stress, *k* represents the steepness of the response curve, and *T*_m_ is the midpoint of the symmetrical curve (an estimate of LT50). Curve fitting was carried out using quantile regression.

Frost tolerance of fine roots was quantified by REL for three populations (DE1, BG, and ES) on 30 January 2011 at the cold site. For this, fine roots of three individuals per population and temperature treatment were washed out and immediately tested according to the protocol described above for buds. Special care was taken to avoid desiccation of the roots.

Winter survival at the cold site was determined per plant in May 2011, when a high share of plants did not develop leaves and dried out completely. At the end of the growing season (8 September 2011), we prepared cross sections of the root collars with a microtome of 14 dead and 22 surviving individuals randomly drawn from all populations and manipulations. Those thin sections were stained according to standard routines (Schweingruber et al. 2006) and analyzed on the computer screen. Measurements of the annual rings were taken on a sliding stage under a light microscope with the program TsapWin (Rinntech, Heidelberg, Germany).

Bud phenology per individual was quantified at the warm site on April 16, both in 2011 and 2012 distinguishing between dormant buds, buds swollen and elongated, and buds broken open with first green visible.

Mean annual and monthly minimum temperatures for the period 1950–2000 (mean temperature of the coldest month for the years 1950–2000) for each geographic origin of the populations were retrieved from WorldClim at a resolution of 5′ (Hijmans et al. 2005) and used as indicators for minimum temperatures. Although these values exceed absolute minimum temperatures considerably due to averaging (for the cold experimental site, mean annual minimum temperature based on WorldClim is −3.5°C, while absolute minimum temperatures between 1997 and 2013 ranged between −10.8 and −25.6°C with the winter of the experiment being the second coldest on record), we assume that the relative differences between geographic origins are adequately captured. We use these surrogates because climate stations are not available in reasonable vicinity to our geographic origins (10 km).

### Statistics

ANOVA in combination with linear mixed models (all numerical response parameters, cold site with nesting as random effect) and generalized linear mixed models (logistic response parameters, nesting as random effect) was applied. The additional climate treatments (warming at the cold site and summer drought at the warm site) were tested as main effects in interaction with the population origin. Local adaptation was tested by linear regressions of the response parameters versus minimum temperatures at the origins of the populations. Here, range (marginal vs. central populations) was used as a random effect in linear mixed models (numerical responses) and generalized linear mixed models (logistic response), and least-squares regressions for both groups were run subsequently in case of significant correlations obtained by the mixed models. Note that no significant correlations were found without accounting for marginal and central origin.

The single and joint influence of the origin (minimum temperature at the origin), bud phenology (phenological stage on 16 April 2012), and pretreatment (drought or control treatment in the preceding summer) on frost tolerance was evaluated by variance partitioning (Legendre 2008). For each explanatory variable, the significance of the optimal relation (i.e., quadratic, square root, log transformation) to the dependent variable was assessed beforehand by univariate linear least-squares regression analysis.

All analyses were run in R (R Development Core Team 2011) with the additional packages vegan 1.17-12 (function varpart), nlme 3.1-103 (function lme), lme4 0.999375-42 (function glmer), quantreg 4.71 (function nlrq), multcomp 1.2-7 (function glht), and raster 1.9-44 and sciplot 1.0-9 for graphical illustrations.

## Results

### Local adaptation, differences between marginal and central populations, and response to warming at the cold site

**Figure 2:**
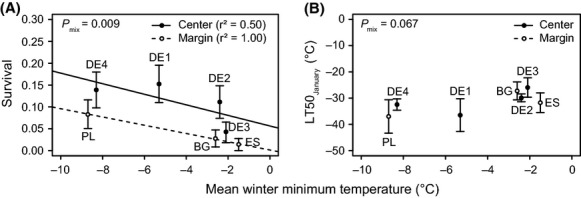
Winter survival (A) and bud frost tolerance (expressed as LT50) (B) at the cold site in relation to the mean winter minimum temperature at the origins of the populations. Mean values and standard errors over all plants from one population are displayed (A: *n *= 72 individuals; B: *n *= 6 mixed samples). The *P*_mix_ values stem from generalized linear mixed-effects (A) and linear mixed-effects (B) models with the temperature treatment and the range (margin vs. central) as random effects.

Only 8.1% of all plants survived the first winter at the cold site. Survival was higher in the warming treatment (15.5%) than in the reference (0.8%; *P *< 0.001). Survival further differed between populations (*P *= 0.002) and showed signs of local adaptations with populations from colder origins showing higher survival than populations from warmer origins (*P*_mix_ = 0.009; Fig. 2A). This pattern was true for marginal (*r*² = 1.00) and central (*r*^2^ = 0.50) populations; marginal populations, however, showed a generally reduced survival along the temperature gradient in comparison with central populations. Killed individuals and survivors showed no difference in the width of the first annual ring (killed: 3.1 ± 0.3 mm SE, survived: 2.9 ± 0.2 mm SE; *P *= 0.614). None of the killed individuals showed signs of a second growth ring, implying that no spring growth occurred. Surviving individuals showed a second growth ring, which was much smaller than their first growth ring (mean: 0.7 mm, see supporting information, Figure S2).

**Figure 3:**
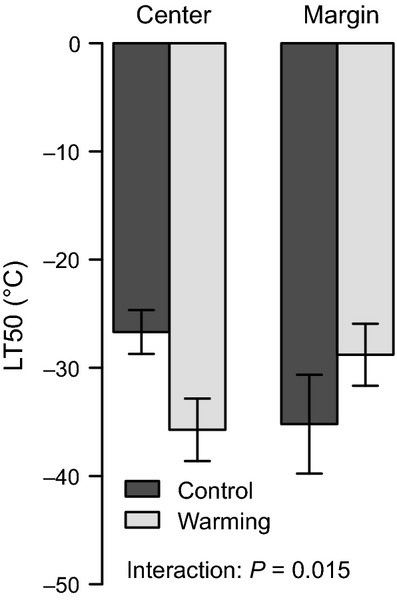
Bud frost tolerance (expressed as LT50) at the cold site as affected by chronic warming and population origin (center vs. margin). Mean values and standard errors are displayed.

Bud frost tolerance expressed as LT50 values of the plants in January did not significantly vary with winter minimum temperatures at the origins of the populations (*P*_mix_ = 0.067; Fig. 2B) or warming treatment (*P*_mix_ = 0.325). Neither populations nor central versus marginal populations differed in their mean bud frost tolerance in mid-winter (Table 2). Variance in bud frost tolerance differed slightly among populations (*P *= 0.046) but not between central and marginal populations (*P *= 0.416). Yet the warming effect on mean LT50 differed between central and marginal populations (interaction: *P *= 0.015). While warming led to increased bud frost tolerance in the central populations, warming reduced bud frost tolerance in the marginal populations (Fig. 3). The mean bud frost tolerance over all plants was LT50 = −31.6°C, and no sign of natural frost damage to the buds was observed.

**Table 2:** ANOVA results for the statistical analyses of the data from the cold site experiment.

	Winter survival	Bud frost tolerance in January (LT50)
Population	**0.002**	0.277
Warming	**<0.001**	0.325
Interaction	0.314	0.066
Model (random effects)	glmer (population, block)	Rank-based lm
Variance across populations (Levene's test)	Binomial	**0.046**
Central vs. marginal	**0.040**	0.874
Warming	**<0.001**	0.344
Interaction	0.314	**0.015**
Model (random effects)	glmer (population, block)	lme (population)
Variance central vs. marginal (Levene's test)	Binomial	0.416

Bold indicates significant effects (*P* < 0.05).

Roots were less frost tolerant (mean LT50 = −15.8°C), again without showing signs of natural frost damage in the REL test. The three tested populations differed in their frost tolerance of the roots (*P *= 0.036) with the central population DE1 showing higher tolerance (mean LT50 = −23.1 ± 4.7°C SE) than the marginal populations ES (−14.1 ± 1.5°C) and BG (−10.1 ± 2.4°C). No significant difference was found between the two marginal populations tested for root frost tolerance. Frost tolerance of the fine roots was not affected by the temperature treatment (*P *= 0.334).

### Local adaptation, differences between marginal and central populations, and response to preceding drought at the warm site

**Figure 4:**
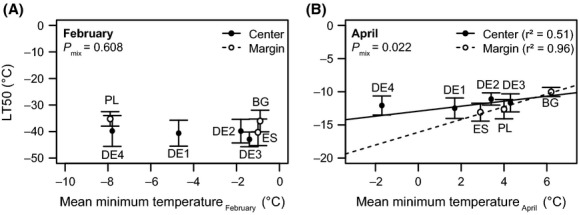
Winter (A) and spring (B) frost tolerance (expressed as LT50) at the warm site in relation to the mean winter minimum temperature at the origins of the populations. Mean values and standard errors over all plants from one population are displayed (*n *= 8). The *P*_mix_ values stem from linear mixed-effects models with the drought pretreatment and the range (margin vs. central) as random effects. Linear regressions per range group are displayed only of *P*_mix_ < 0.05.

Frost tolerance in mid-winter at the warm site was not significantly related to minimum temperature at the origins (Table 3; Fig. 4A). Here, all plants survived both winters, and the mean frost tolerance in the second winter reached LT50 = −39.3°C.

**Table 3:** ANOVA results for the statistical analyses of the data from the warm site experiment.

	Bud frost tolerance in February (LT50)	Bud frost tolerance in April (LT50)	Buds dormant 16 April 2012
Population	0.867	0.657	0.479
Drought	0.413	**0.014**	**0.038**
Interaction	0.730	0.354	0.381
Model (random effects)	lm	lm	glmer
Variance across populations (Levene's test)	0.946	0.604	Binomial
Central vs. marginal	0.277	0.904	0.255
Drought	0.380	**0.015**	0.060
Interaction	0.173	0.830	0.336
Model (random effects)	lme (population)	lme (population)	glmer (population)
Variance central vs. marginal (Levene's test)	0.441	0.992	Binomial

Bold indicates significant effects (*P* < 0.05).

Frost tolerance in April, however, was related to April minimum temperatures at the origins (*P*_mix_ = 0.022; Fig. 4B). A significant linear regression between frost tolerance and minimum temperature at the origin was found only for the marginal populations (*r*^2^ = 0.96) and not for the central populations (*r*^2^ = 0.51). The mean frost tolerance in April reached LT50 = −11.9°C.

**Figure 5:**
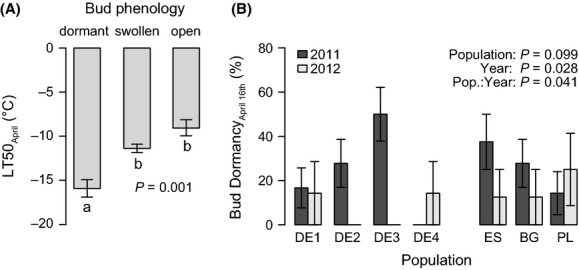
Spring frost tolerance depended on bud phenology (A). Frost tolerance, expressed by LT50, and bud phenology were determined on 16 April 2012. Significance according to linear mixed model with pretreatment as random effect. Lowercase letters indicate homogenous groups according to TukeyHSD post hoc comparisons. Populations differ in their share of plants with dormant buds on April 16 over the two study years (B). Displayed are mean values and standard errors over 8 plants per bar. ANOVA results of generalized linear mixed models on a logistic response (dormant yes/no) with pretreatment as random effect are displayed.

Spring frost tolerance depended on bud phenology (*P *= 0.001): Open and swollen buds showed a mean reduction in frost tolerance by 5.7°C in comparison with dormant buds, while the former two stages did not differ significantly from each other (Fig. 5A). The origins differed in the share of individuals with dormant buds in April between the two study years (*P *= 0.041; Fig. 5B). This significant interaction between origin and year implies that differences in bud phenology among populations were altered from the first to the second year. This divergence between the 2 years is clearly visible in the central populations, while the marginal populations tended to respond more stable (Fig. 5B).

**Figure 6:**
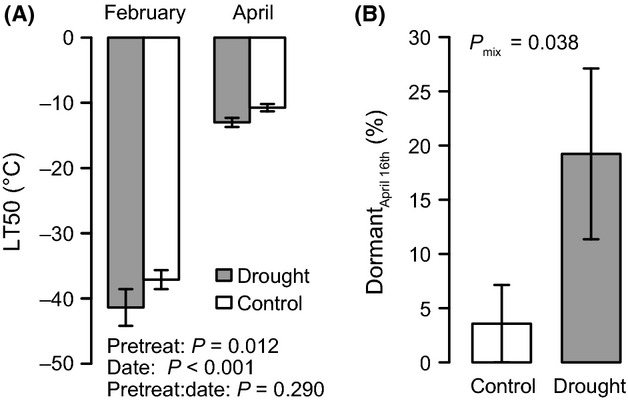
Exposure to drought in the preceding summer affects frost tolerance, expressed as LT50 values, in winter and spring (A) and bud phenology, expressed as share of plants with dormant buds on April 16 (B). ANOVA results of linear mixed models (A) and generalized linear mixed models on a logistic response (dormant yes/no) (B) with population as random effects are given. Mean values and standard errors are displayed with *n *= 28 plants per bar.

Summer drought in the preceding year increased the frost tolerance of the plants (*P *= 0.014; Fig. 6A). This effect was stable across winter and spring (interaction between pretreatment and date: *P *= 0.290). Surprisingly, bud penology was also affected by the drought pretreatment: Plants that had experienced drought started bud break later than those not exposed to drought in the preceding summer (*P *= 0.038; Fig. 6B).

**Figure 7:**
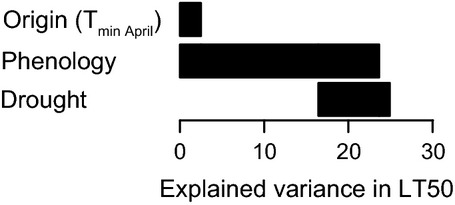
Explained variance in spring frost tolerance (LT50) of two-year-old *F. sylvatica* seedlings by the April minimum temperature at the origin of the populations, phenological stage of the buds on April 16, and exposure to drought in the preceding summer. Correlated variables can explain the same variance, the applied variance partitioning therefore distinguishes between individually (no vertical overlap of bars) and jointly (vertical overlap of the bars) explained variance.

Spring frost tolerance was mainly driven by bud phenology, which independently explained 14% of the variance in frost tolerance and another 10% jointly together with the pretreatment and the April minimum temperature at the origin of the populations (variance partitioning; Figure 7). The climatic origin and the pretreatment individually explained only negligible parts of the variance, implying that they acted mainly through changes in phenology.

## Discussion

### Local adaptation

Frost tolerance of juvenile *F. sylvatica* depended on the climatic origin of populations in this study, with inferior bud frost hardiness in spring and reduced winter survival for populations from warmer climates. This result implies local adaptation to winter and late spring frost in the studied populations of *F. sylvatica*, which is in line with previous findings for our target species (Visnjic and Dohrenbusch 2004; Czajkowski and Bolte 2006; Kreyling et al. 2012a) and other tree species (Kuser and Ching 1980; Saenz-Romero and Tapia-Olivares 2008; Kreyling et al. 2012c). This finding further emphasizes the ecological and evolutionary importance of short-term thermal events such as frost (Inouye 2000). The local adaptations, however, became significant only when the models accounted for the difference between central and marginal populations, thereby indicating the importance of the biogeographical setting of populations within the species range.

Marginal populations with regard to the species range showed stronger local adaptation with lower survival (Fig. 2) and higher slope in the linear regression between LT50 and climate at the origin in spring (Fig. 4B) along the climatic gradient of population origins than populations from the center of the species' range. Length of the climatic gradient was very similar for both groups. Despite low population numbers in this study, this finding corresponds to expectations and can be explained by a stronger selective pressure of environmental conditions and limited genetic exchange in geographically isolated marginal populations (Choler et al. 2004; Kawecki 2008; Paul et al. 2011). While there is a multitude of studies on differentiation among populations in forest trees (provenance trials), case studies on local adaptations differentiating among central and marginal populations are rare. However, there is evidence for superior performance of a marginal population in its native environment versus worst performance of this population in other environments in comparison with populations from the range center (Coulleri 2010). Such local adaptations may contribute to the extension of species ranges, as they would expand the ecological niche of the species (Kawecki and Ebert 2004; Holt and Barfield 2011). Examples such as ours or the one of Coulleri (2010) provide an indication that local adaptations can be particularly strong in marginal populations. Therefore, the so-called swamping gene flow hypothesis, that is, the asymmetrical gene flow from large, presumably well-adapted central populations toward small, marginal populations (Paul et al. 2011), does not prevent marginal populations from local adaptation. It is suggested that rapid climate change may pose threats to locally adapted marginal populations (Nunes et al. 2009). Conversely, selection and reduced gene flow in marginal populations may also enable relatively quick adaptations to changing climate even in slow-growing forest trees (Jump et al. 2006). Insights from invasion ecology suggest that small population sizes can enable quick adaptations in some cases while ending fatal for the populations in the majority of cases (Sax et al. 2007). Detailed understanding of local adaptations in marginal populations is therefore highly relevant for the understanding of species ranges and range shifts in changing environments. Here, within-population variability requires attention. Due to enormous seed loads and self-thinning after establishment, this high variation within populations, which is expressed by large standard errors in all figures, can be expected to foster selection and adaptation, thereby determining genetic constitution of future stands (Hosius et al. 2006).

Here, we considered two marginal populations from the trailing edge and one (PL) from the leading edge of the species distribution. We lump those three together based on the notion that genetic diversity within populations is generally expected to be reduced in marginal populations, no matter if located at the trailing or leading edge, while differentiation among populations is higher at the trailing edge (Hampe and Petit 2005).

Our study and many other common garden experiments (e.g., Kuser and Ching 1980; Joshi et al. 2001; Hufford and Mazer 2003; McKay et al. 2005; Bennie et al. 2010; Kreyling et al. 2012a,b2012b; Thiel et al. 2014) imply that there are considerable differences among populations in their adaptations to environmental drivers. Climate change will therefore require range shifts or adaptation of populations throughout the range of the species, not only at the southern trailing edge. If frost is a limiting factor for the full species range (Bolte et al. 2007), we expect that this can also be the case for southern populations migrating northwards with climate warming both, naturally or through assisted migration. Frost events becoming less frequent but unchanged in magnitude and duration (Kodra et al. 2011) will still occur very probably within the life of single-tree individuals.

### Winter versus spring frost tolerance

Differences in late spring bud frost tolerance between populations were stronger than differences in mid-winter bud frost tolerance at the warm experimental site. The survival and frost tolerance data from the cold site, however, indicated that any differences, for example, in frost tolerance of other organs in mid-winter, can become crucial for the survival of individuals. Based on the minimum temperatures in the soil and close to the plants, we suggest that embolism damaged the transport systems already early in the winter when temperatures dropped to −19.1°C in control and −15.8°C in the warming treatment on 30 December 2010. Buds survived these temperatures as they showed reasonable LT50 values at the end of January. In spring, however, the damaged transport systems proved fatal for the whole plant, and no radial growth at the onset of spring was detected for the killed individuals. Even the surviving individuals showed very little radial growth in the second year in comparison with the first year (−75%), which indicates that they also suffered from frost damage. Winter frosts with temperatures below −17°C reportedly lead to mortality of juvenile *F. sylvatica* (Bolte et al. 2007), and there is evidence for winter embolism leading to failure of xylem activation in spring in this species (Coleftd et al. 2001). The deciduous habit of the target species renders frost drought in juvenile plants, although recently transplanted, as alternative explanation for the observed mortality less likely.

Variation in winter frost tolerance can be explained by different resource allocation toward cryoprotection of living cells (Morin et al. 2007; Kreyling et al. 2012c) or differential tolerance and recovery from embolism, for example, by wood anatomical features (Martin et al. 2010). While both processes could also play a role in frost tolerance of the buds in spring, our data imply that variation in spring frost tolerance can mainly be attributed to phenological differences (Fig. 7). Earlier onset of the vegetation period with climate warming (e.g., Parmesan 2007) in combination with no change in the duration and magnitude of minimum temperature extremes (Kodra et al. 2011), however, may lead to increasing risk of late frost damage in plants (Augspurger 2013). With regard to this dilemma, it is interesting that the populations in our study showed differences in their bud phenology in response to the two study years (Fig. 5B). This implies that cues for bud development differ among the populations. A stable phenology over the years points at photoperiod as the main driver, while variation in phenology may hint at preceding temperature conditions being the dominant cue. Late-successional tree species are generally known to rely on photoperiod while early-successional species show stronger temperature dependence in their bud phenology (Basler and Koerner 2012). However, variation in bud break of *F. sylvatica* seedlings in a common garden experiment is attributed to different temperature sum requirements (von Wuehlisch et al. 1995).

Bud frost tolerance, however, did not reflect such differences among populations. LT50 values measured for the buds in mid-winter exceeded realized temperature minima considerably. Similar levels of frost tolerance are reported for *F. sylvatica* by Leftra-Vaskou et al. (2012) and Lenz et al. (Armando Lenz, Basel, pers. communication). As indicated in the methods section, we refer from further interpretation of the absolute values as they depend strongly on the protocol for measuring REL and are consequently hardly comparable among studies.

### Continuous warming and winter frost tolerance

We expected reduced frost tolerance with continuous warming (Eccel et al. 2009). Yet no general reduction in frost tolerance expressed as LT50 values was observed in our warming manipulation at the cold site. Surprisingly, frost tolerance was even increased by warming in the central populations, while it tended to decrease in the marginal populations (Fig. 3). While this finding emphasizes the difference among central and marginal populations, we cannot explain it with the available data. Clearly, phenotypic plasticity in central and marginal populations requires further consideration.

### Preceding water stress affects frost tolerance

In accordance with previous findings (Blodner et al. 2005; Kreyling et al. 2012c), water stress in the preceding summer increased frost tolerance in winter by 4.3°C and in spring by 2.3°C. This can be explained by the mechanistic similarity of frost and drought stress avoidance, which both aim at prevention of intracellular dehydration and stabilization of cell membranes by accumulation of a wide range of carbohydrates (Beck et al. 2007). The observed differences in spring frost tolerance after experience of drought stress were linked to delayed bud break in our study. Again, this is surprising as phenological shifts in response to extreme drought are reported to be most important for those phenological events, which happen closely after the drought (Nagy et al. 2013). Understanding memory effects and delayed responses is therefore important for our ability to project species responses to climate change (Walter et al. 2013).

## Conclusions

Seedlings of *F. sylvatica* showed evidence for local adaptations to winter and spring frosts which were stronger developed in three marginal than in four central populations referring to the range of the species. This finding generally emphasizes the ecological and evolutionary importance of frost and of winter processes. Intraspecific variability and local adaptations appear crucial for range limits, as they broaden the ecological niche of a species. We confirmed that preceding summer drought can improve frost tolerance and found no difference in the sensitivity of populations in this drought effect. The response of bud frost tolerance in winter to continuous warming, however, differed between central and marginal populations, thereby raising the question how marginal populations will be able to adapt to ongoing climate change.

## Supporting Information

**Figure S1.** Air temperature at the two experimental sites over the course of the experiments. **Figure S2.** Cross section of one individual which survived winter at the cold site.
