# Supplementary material for: Local adaptations to frost in marginal and central populations of the dominant forest tree Fagus sylvatica L. as affected by temperature and extreme drought in common garden experiments
Source: Ecol Evol. 2014 Feb 7;4(5):594–605. doi: 10.1002/ece3.971 (PMC4098140; doi:10.1002/ece3.971)
Supplement: Supplementary file 1 — Figure S1. Air temperature at the two experimental sites over the course of the experiments. Figure S2. Cross section of one individual which survived winter at the cold site. [file ece30004-0594-sd1.docx]

**Supporting information**


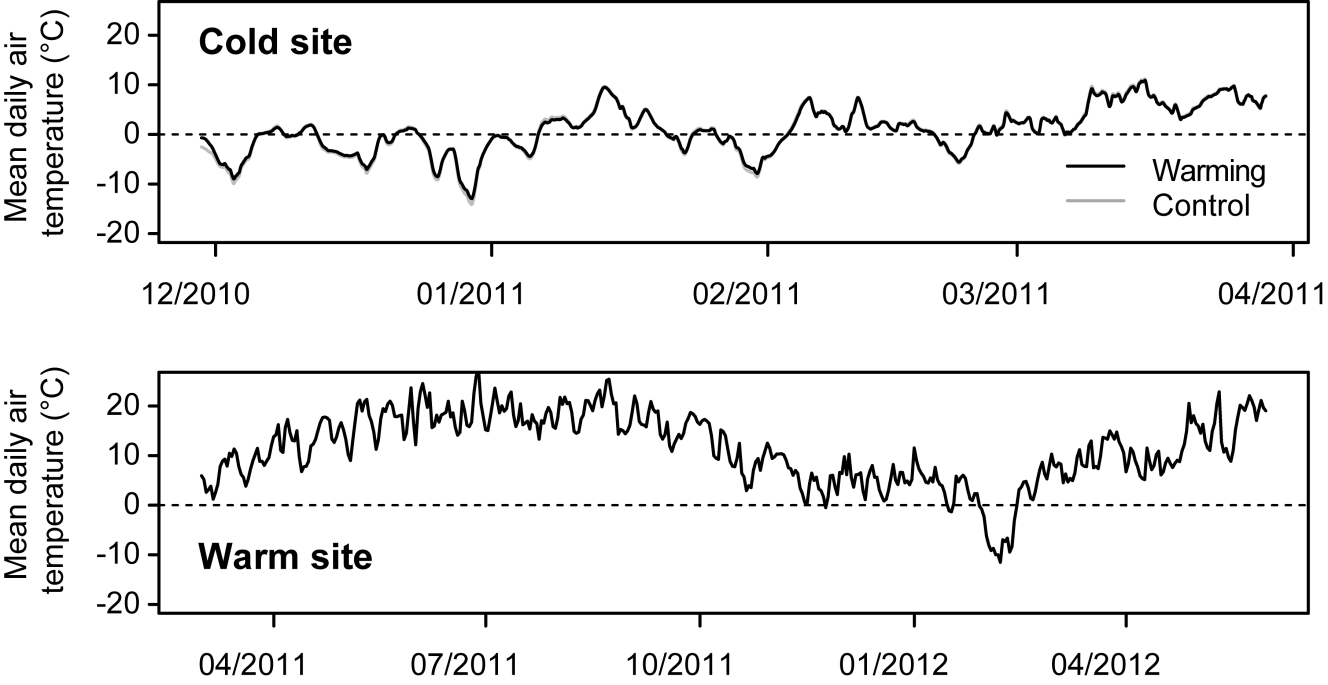


Figure S1: Air temperature at the two experimental sites over the course of the experiments. Displayed are running means over 24h (cold site) or daily mean temperatures (warm site). Minimum air temperatures reached -18.6°C in the control and -17.1°C in the warming treatment at the cold site at December 30^th^, and -15.2°C (February 7^th^) at the warm site.


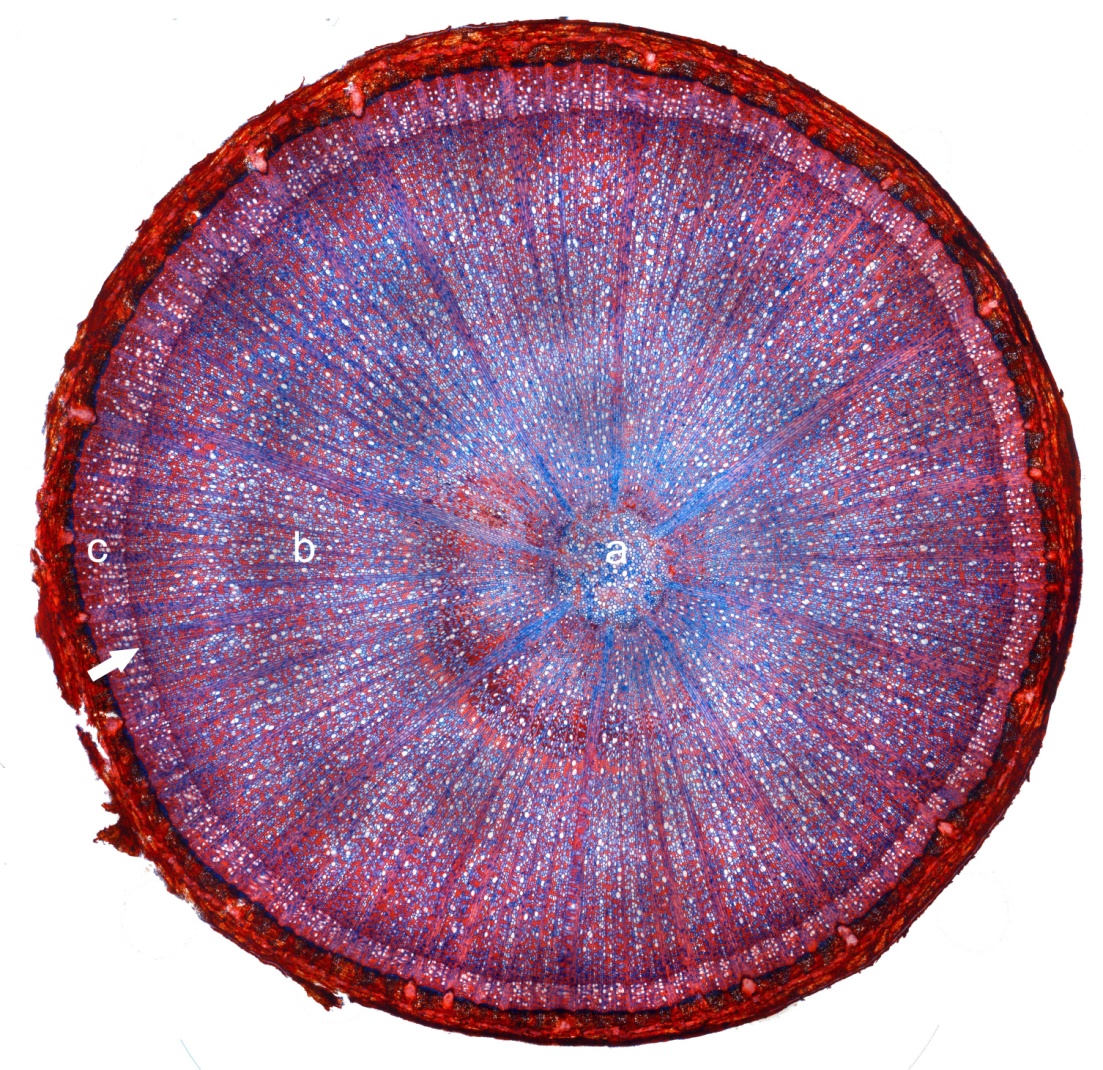


Figure S2: Cross section of one individual which survived winter at the cold site. Lower case letters indicate years of growth, the arrow marks the start of growth in the year after the severe winter frost killed many other individuals at the cold site.
